# Supplementary material for: L-Glyceraldehyde Inhibits Neuroblastoma Cell Growth via a Multi-Modal Mechanism on Metabolism and Signaling
Source: Cancers (Basel). 2024 Apr 25;16(9):1664. doi: 10.3390/cancers16091664 (PMC11083149; doi:10.3390/cancers16091664)
Supplement: Supplementary file 1 [file cancers-16-01664-s001.zip › Supp_figure1C_2.pdf]

Supplementary data for figure 1C

GI-M-EN

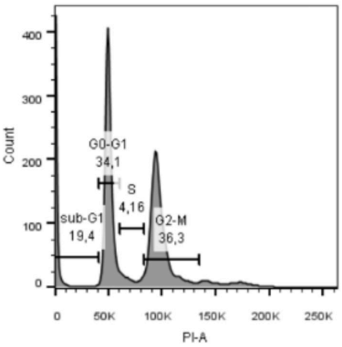

DBPS 72h_Tube_001.fcs
Single Cells
8422

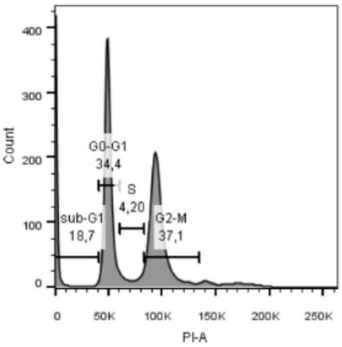

DBPS 72h_Tube_002.fcs
Single Cells
8224

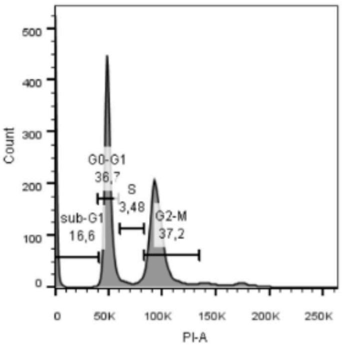

DBPS 72h_Tube_003.fcs
Single Cells
8169

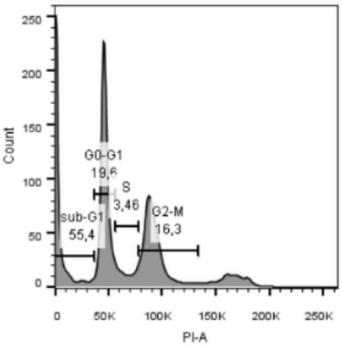

L-GA 72h_Tube_001.fcs
Single Cells
9126

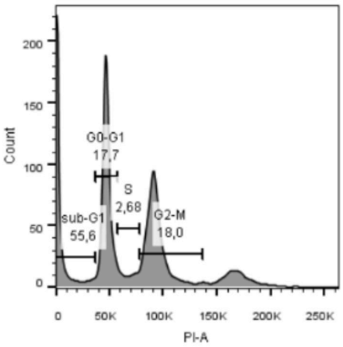

L-GA 72h_Tube_002.fcs
Single Cells
8736

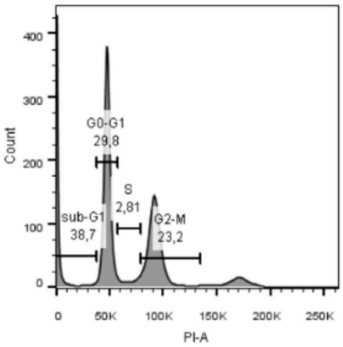

L-GA 72h_Tube_003.fcs
Single Cells
8929

SK-N-AS

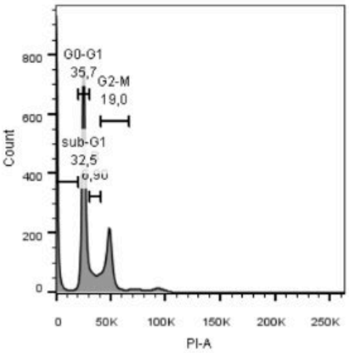

DBPS 72h_Tube_001.fcs
Single Cells
9454

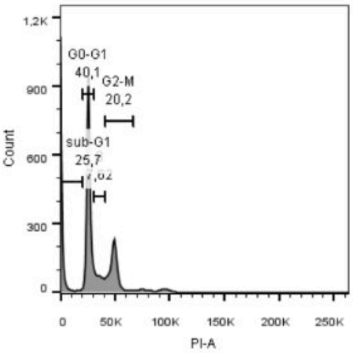

DBPS 72h_Tube_002.fcs
Single Cells
9445

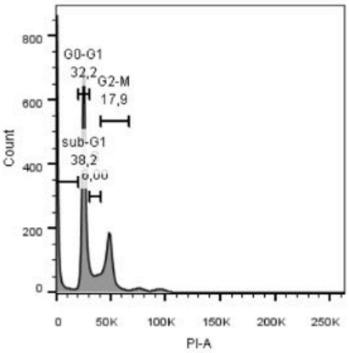

DBPS 72h_Tube_003.fcs
Single Cells
9406

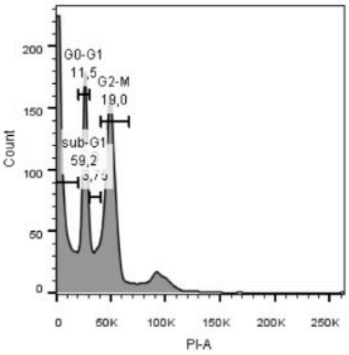

L-GA 72h_Tube_001.fcs
Single Cells
9566

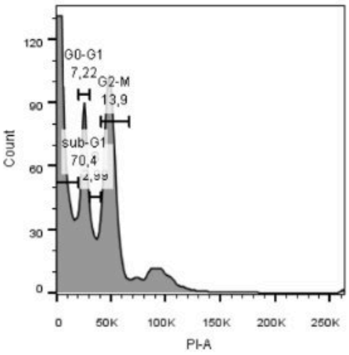

L-GA 72h_Tube_002.fcs
Single Cells
9632

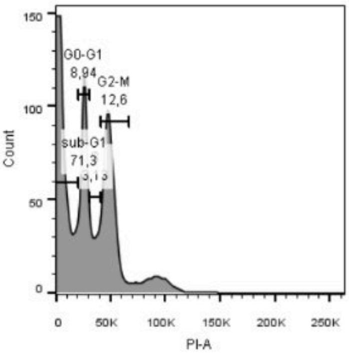

L-GA 72h_Tube_003.fcs
Single Cells
9590
