# Supplementary figures and images for: L-Glyceraldehyde Inhibits Neuroblastoma Cell Growth via a Multi-Modal Mechanism on Metabolism and Signaling
Source: Cancers (Basel). 2024 Apr 25;16(9):1664. doi: 10.3390/cancers16091664 (PMC11083149; doi:10.3390/cancers16091664)

# SH-SY5Y

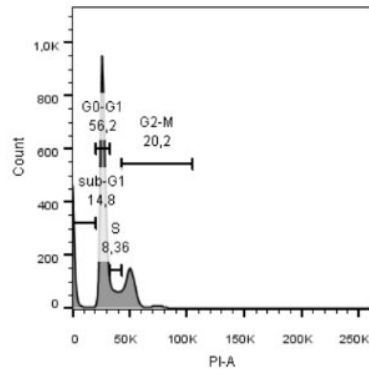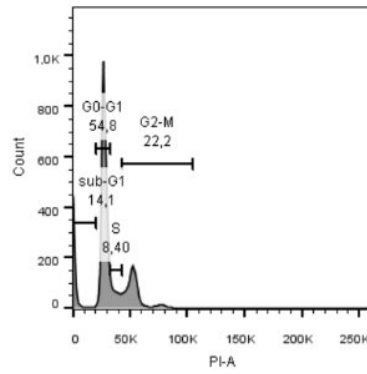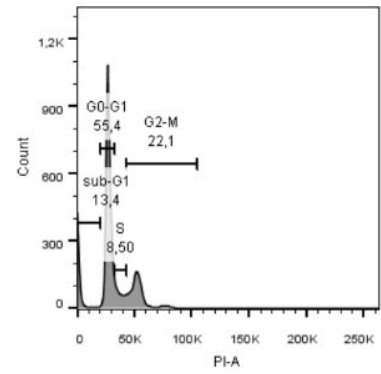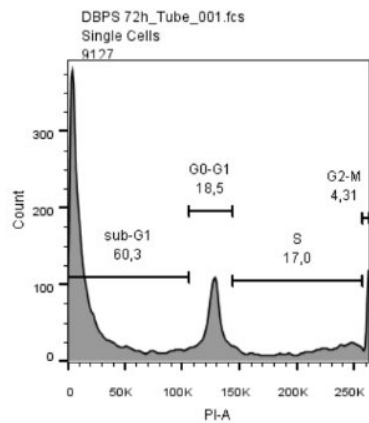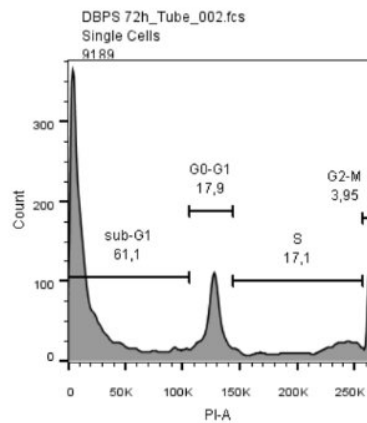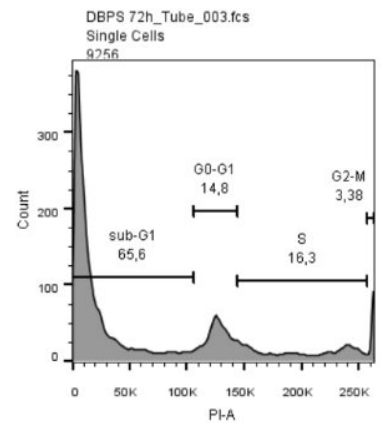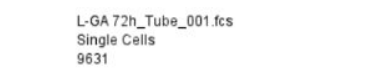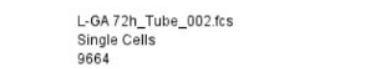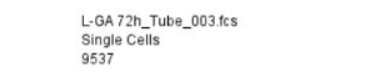

# VH-7

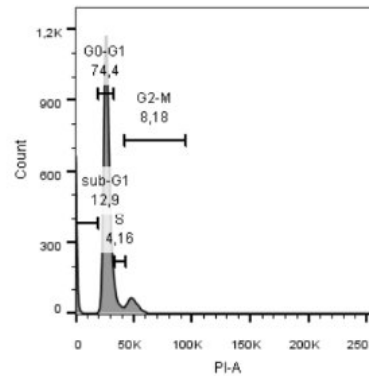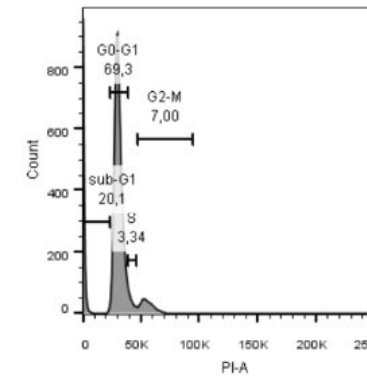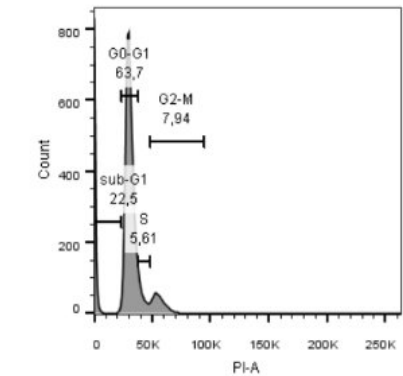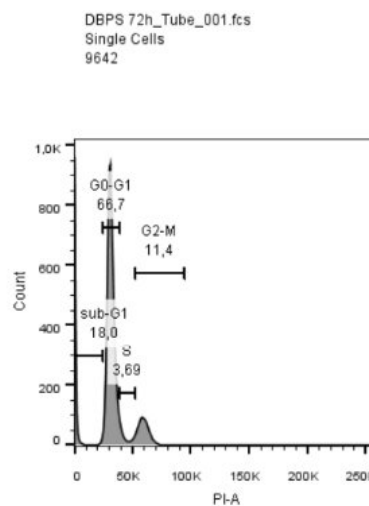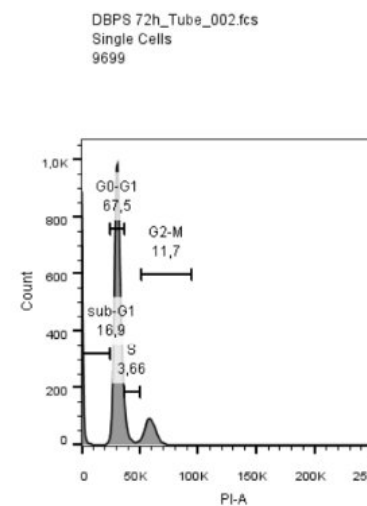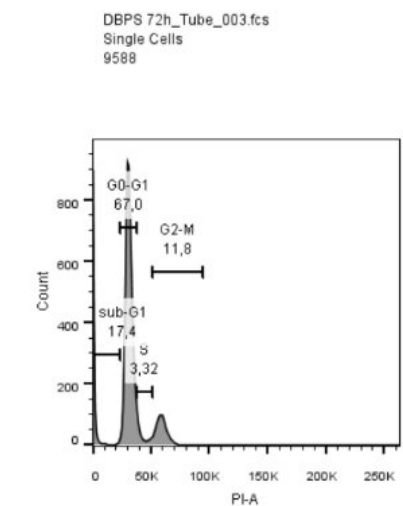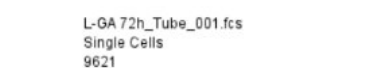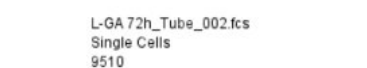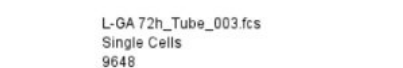

Supplement: Supplementary file 1 [file cancers-16-01664-s001.zip › Supp_figure1C_1.pdf]

CLB-GA

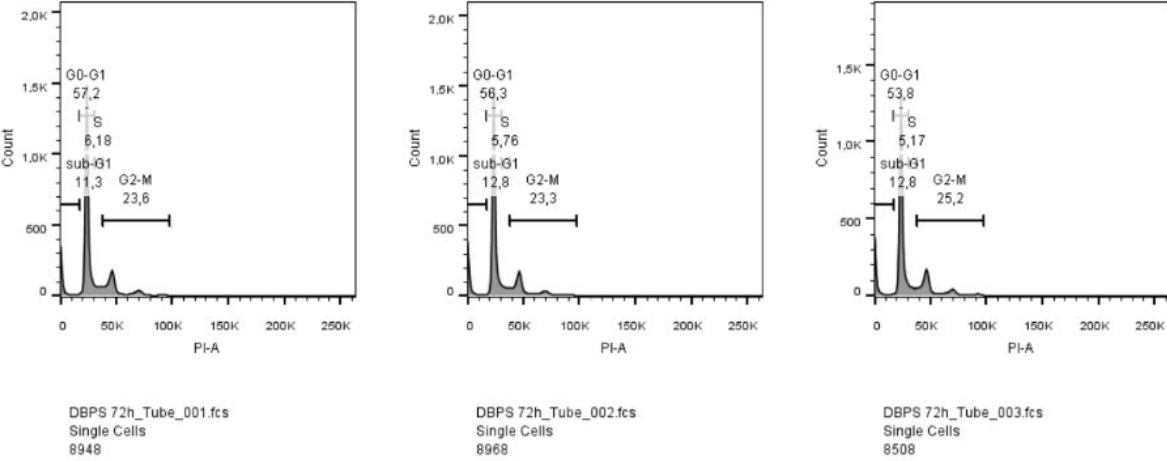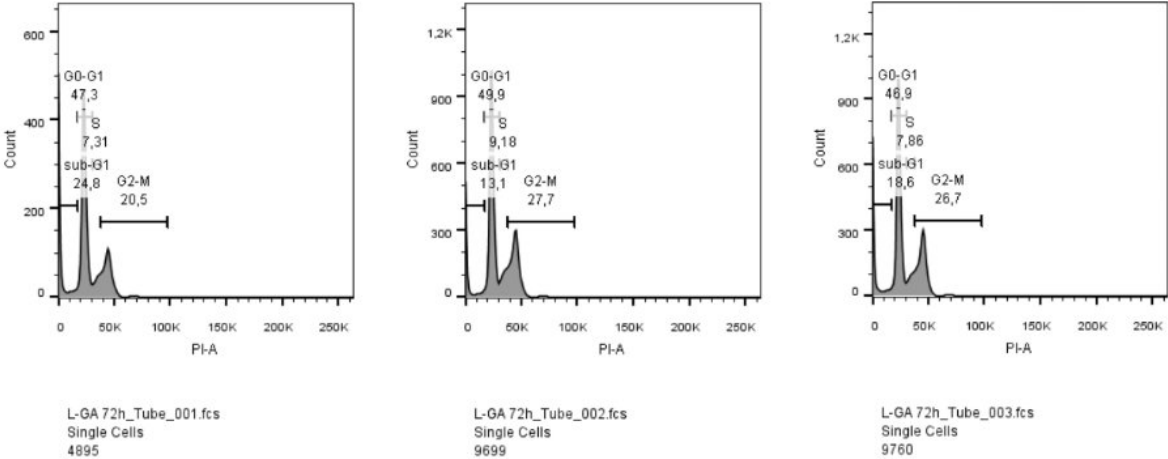

IMR5/75

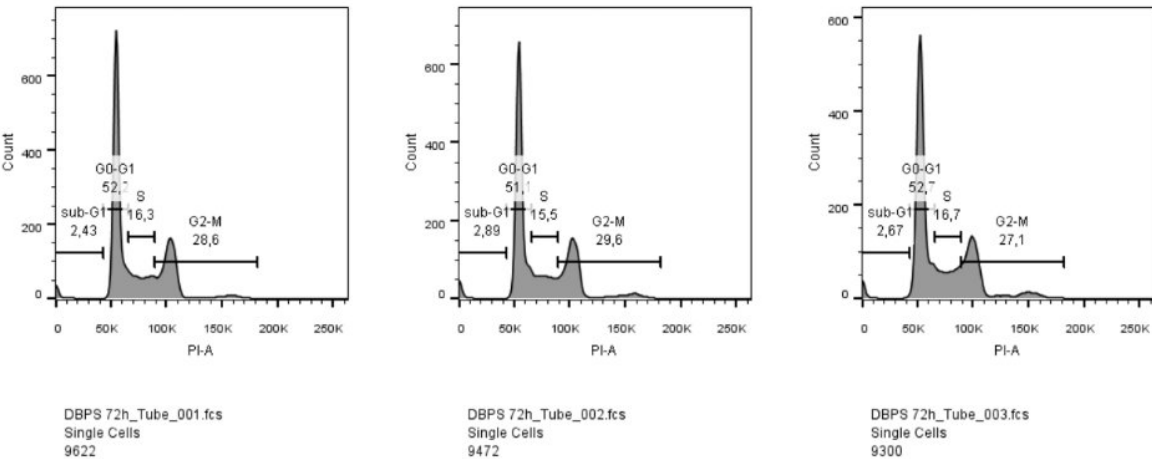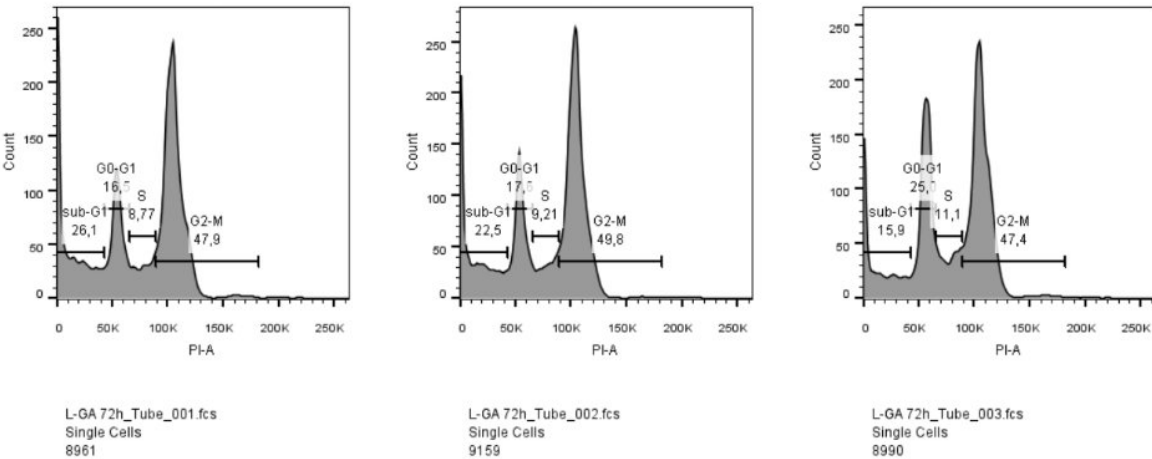

Supplement: Supplementary file 1 [file cancers-16-01664-s001.zip › Supp_figure1C_3.pdf]

BE(2)-C

PBS

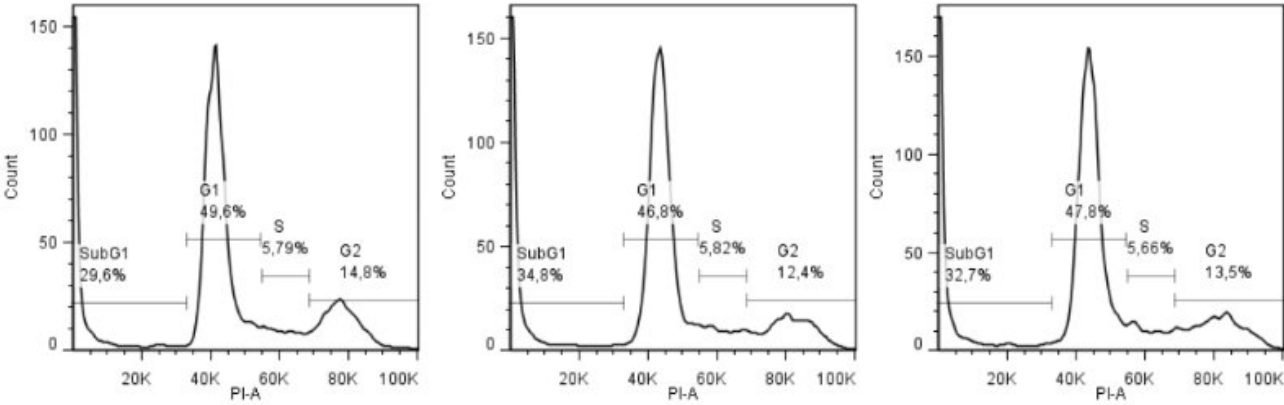

L-GA

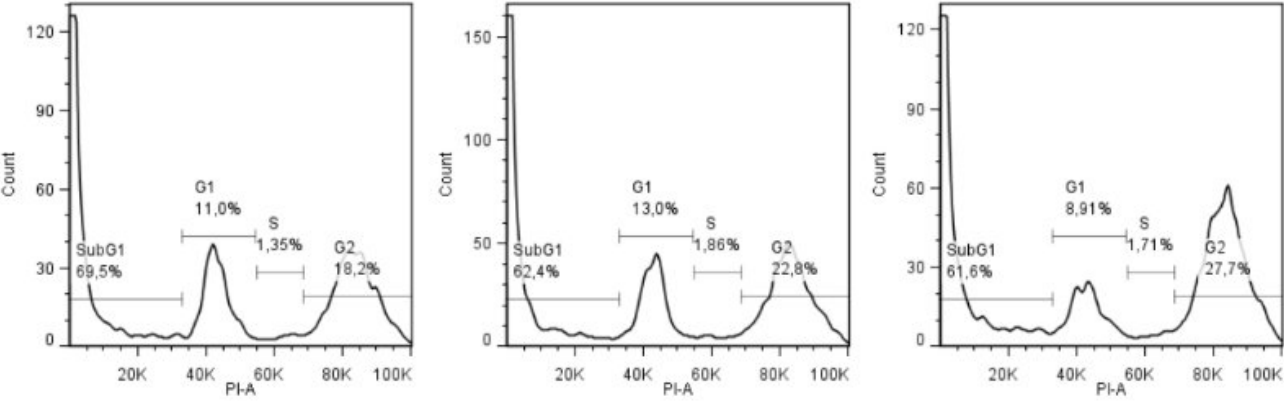

Supplement: Supplementary file 1 [file cancers-16-01664-s001.zip › Supp_figure1C_4.pdf]
